# Development of a root-zone temperature control system using air-source heat pump and its impact on the growth and yield of paprika

**DOI:** 10.1093/aobpla/plae047

**Published:** 2024-09-14

**Authors:** Jeesang Myung, Meiyan Cui, Byungkwan Lee, Hyein Lee, Jaewook Shin, Changhoo Chun

**Affiliations:** Department of Agriculture, Forestry and Bioresources, Seoul National University, 1 Gwanak-ro, Gwanak-gu, Seoul 08826, South Korea; Research Institute of Agriculture and Life Sciences, Seoul National University, 1 Gwanak-ro, Gwanak-gu, Seoul 08826, South Korea; Department of Agriculture, Forestry and Bioresources, Seoul National University, 1 Gwanak-ro, Gwanak-gu, Seoul 08826, South Korea; Department of Agriculture, Forestry and Bioresources, Seoul National University, 1 Gwanak-ro, Gwanak-gu, Seoul 08826, South Korea; Department of Agriculture, Forestry and Bioresources, Seoul National University, 1 Gwanak-ro, Gwanak-gu, Seoul 08826, South Korea; Department of Agriculture, Forestry and Bioresources, Seoul National University, 1 Gwanak-ro, Gwanak-gu, Seoul 08826, South Korea; Research Institute of Agriculture and Life Sciences, Seoul National University, 1 Gwanak-ro, Gwanak-gu, Seoul 08826, South Korea

**Keywords:** Energy efficiency, fruit yield, greenhouse cultivation, nutrient solution temperature, paprika, root-zone temperature

## Abstract

By developing and implementing a local temperature control system, such as a root zone, with a high energy efficiency heat source, we can ensure both yield and energy efficiency against extreme temperatures. This system, designed with practicality in mind, has a remarkably positive impact on paprika plants’ growth and yield in greenhouse cultivation. In the summer season, paprika plants were grown with no cooling, nutrient solution cooling (NSC) and the combination of NSC and substrate surround cooling (SSC) (NSC + SSC). In the case of SSC, cooled water circulated through the pipe surrounding the substrate to lower the substrate temperature. The cooling system maintains the nutrient solution temperature at 18 °C and the circulating water temperature at the system in the winter season; the paprika plants were grown with no heating (NH), nutrient solution heating (NSH) and the combination of NSH and substrate surround heating (SSH) (NSH + SSH). The heating system maintains the nutrient solution temperature at 25 °C and the circulating water temperature at 30 °C. In the summer, the root fresh and dry weights, stem fresh and dry weights, stem length and node number were increased in the NSC + SSC. In the winter season, the stem fresh and dry weights, leaf area and leaf fresh and dry weights were increased in the NSH + SSH. In both seasons, root-zone temperature control increased the fruit quality and yield. The result indicates that this easy-to-install root-zone temperature control system can be applied to the commercial greenhouse to secure paprika growth and yield in year-round cultivation.

## Teaser

The root-zone temperature control system, which uses the high energy efficiency heat source, the air-source heat pump, was developed in this research. The developed root-zone temperature control system can be easily applied to the commercial greenhouse and used to counteract the extreme temperature of the summer and winter seasons, respectively. The control of the root-zone temperature can secure paprika growth and yield in year-round cultivation by increasing nutrient uptake and decreasing temperature stress.

## Introduction

In greenhouse paprika cultivation, plants can suffer from extremely high and low temperatures in the summer and winter seasons, respectively. This extreme temperature can make the aerial and underground temperatures outside the optimum range and negatively affect paprika plants’ growth and yield. Although there are differences depending on the region, greenhouse temperature control to overcome extreme temperature is associated with significant costs of energy, which accounts for 95 % of energy use of greenhouse operations in southern Europe (Paris *et al.* 2022), 50 % in the southwest USA (Rorabaugh 2015), and 75 % in the northern USA (Runkle and Both 2011).

Local temperature control is one of the powerful tools that can maintain the plant status in the optimal range and simultaneously decrease the energy use in temperature control. When controlling the local temperature inside the greenhouse, selecting the target that can affect the plant effectively is important. Shoot and root meristems, flowers and fruits are known to be temperature sensitive so they can be candidates targets of local temperature control. Shoot-tip and flower temperature control were examined on tomatoes by hanging the heating pipe above the plant canopy (Kawasaki *et al.* 2011). The shoot-tip and flower temperature control systems have disadvantages in adjusting the position according to the plant growth and may block the light supplied to the plant. The root meristem has advantages in being a suitable organ to control the temperature separately because it has a fixed position and is surrounded by the substrate throughout the cultivation period (Peacock 1975). When root-zone temperature increases, root respiration (Ityel *et al.* 2014) and activity (Choi *et al.* 2014) can decrease, and high root-zone temperatures can also decrease leaf photosynthesis (Lee *et al.* 2022). Controlling the root-zone temperature can mitigate plant damage caused by high root-zone temperatures but also that caused by high ambient temperatures (Kuroyanagi and Paulsen 1988; Sasaki and Itagi 1989). Low root-zone temperatures also can negatively affect plant growth and transpiration (Ali *et al.* 2000). Low root-zone temperatures can critically affect the early morning and just before sunset (Abdel-Mawgoud *et al.* 2005). Root-zone heating at these times can promote growth and yield by increasing water and nutrient uptake (Shin *et al.* 2023). Due to these reasons, when the root-zone temperature is managed properly, the total plant growth and the fruit yield can increase by increasing the root activity and alleviating the temperature stress (Rylski and Spigelman 1982; Behboudian *et al.* 1994).

The nutrient solution temperature control systems are partially installed in commercial greenhouses to manage the root-zone temperature. However, clear limitations could be encountered regarding operational scheduling, as irrigation timing must be adjusted based on plant water composition rather than substrate temperature. Thus, the root-zone temperature control system, which can modulate the surrounding temperature and change the substrate temperature directly, is needed to control the root-zone temperature precisely.

When developing the root-zone temperature control system, it is required to introduce a high-efficiency heat source into a greenhouse, to achieve high energy efficiency. Vapour-compressed air-source heat pump systems are suitable for introduction as an energy-efficient heat source due to their simple structure and low initial cost (Zhang *et al.* 2018). Heat pump systems recover heat from a variety of sources for use in a variety of industries and are a key component of energy recovery systems with great energy savings potential. This system has been applied practically and has shown the potential to significantly save energy use in space temperature control by reducing electricity usage by converting and using energy from external heat sources such as air and water (Chua *et al.* 2010; Gaur *et al.* 2021).

In this study, the heat pump-based root-zone temperature control system, which can control the substrate temperature directly by heat conduction, was developed to respond to climate change with high energy use efficiency. The developed root-zone temperature control system can be used alone or as part of a temperature control system and can be an effective and energy-efficient system to respond to extremely high and low temperatures in a greenhouse. To confirm the effectiveness and evaluate the industrial applicability of newly developed root-zone temperature control systems, this study examined the effects of nutrient and root-zone temperature control on the summer and winter season cultivation of paprika in a greenhouse. The effects on the growth, morphology, fruit yield and nutrient uptake were examined.

## Materials and Methods

### Plant materials and environmental conditions

In the summer season, seeds of *Capsicum annuum* Scirocco were sown in a rockwool plug (*ø* 20 mm × H 27 mm; CultiOne S-240, Saint-Gobain Cultilene, Rijen, The Netherlands) on 30 April 2022. The seedlings were grown for 46 days at the greenhouse located in Gangjin (E 126.8°, N 34.6°) and then transplanted on the coir slabs (crushed chips:dust = 5:5 (v/v), 1000 × 150 × 100 mm; Power, Daeyoung GS Co., Daegu, South Korea) to a greenhouse located at Seoul National University in Suwon, South Korea (E 127.0°, N 37.3°) on 15 June 2022. To transplant seedlings that can withstand the high temperature inside the greenhouse, the seedlings were grown 18 days longer before transplanting compared to the winter season. The paprika plants were fertigated by the modified Hoagland nutrient solution (Hoagland and Arnon 1950) at an electrical conductivity of 3.0 and pH of 6.0. For each treatment, 24 plants were transplanted and separated into 3 groups by the position inside the greenhouse and the plants were randomly picked for data analysis.

In the winter season, paprika seeds were sown on 15 September 2022 with the same cultivation materials. The seedlings were grown for 28 days at the same greenhouse as the summer season and transplanted on 11 October 2022. The irrigation was acted every hour from 8:00 to 17:00, and the irrigation time was gradually increased from 2 min at the start of the cultivation to 10 min with the paprika growth.

### Root-zone temperature control

**Figure 1.**
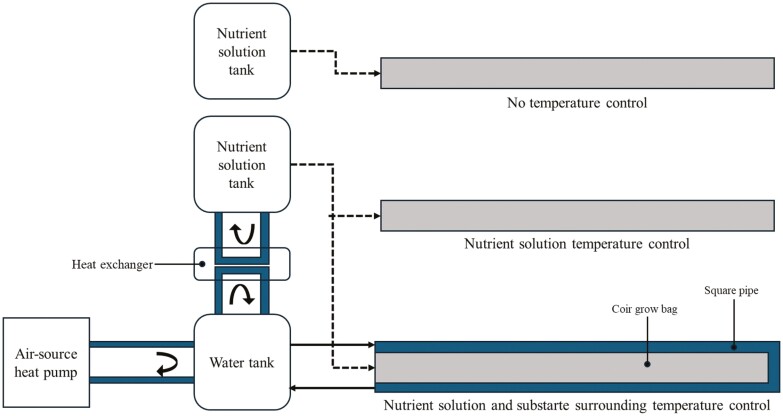
Simplified diagram of the root-zone temperature control systems in the experiment.

The root-zone temperature control system controls the temperature of the nutrient solution and surrounding substrate by using the air-source heat pump (ECO A-05, Innergie Technologies Co., Gwangju, South Korea). When the system operates, the air-source heat pump controls the water temperature inside the tank and the water circulates through the square pipe surrounding the substrate. The water is also used to control the nutrient solution temperature through the heat exchanger installed between the water tank and the nutrient solution tank (Fig. 1).

**Figure 2.**
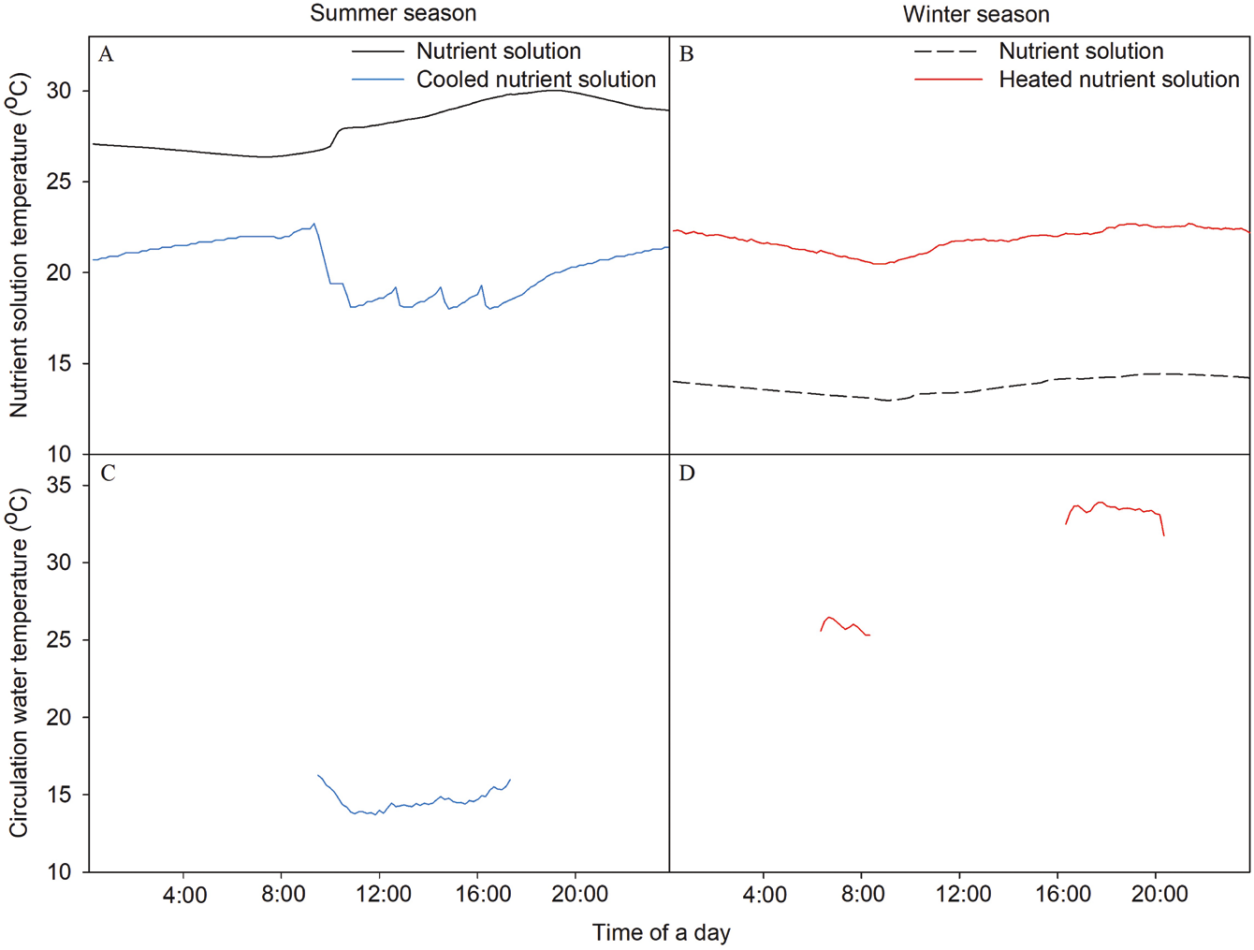
Average temperature of nutrient solution at Control and NSC (A), coolant at SSC (C) in the summer season, nutrient solution at Control and NSH (B) and coolant at SSH (D) in the winter season.

**Figure 3.**
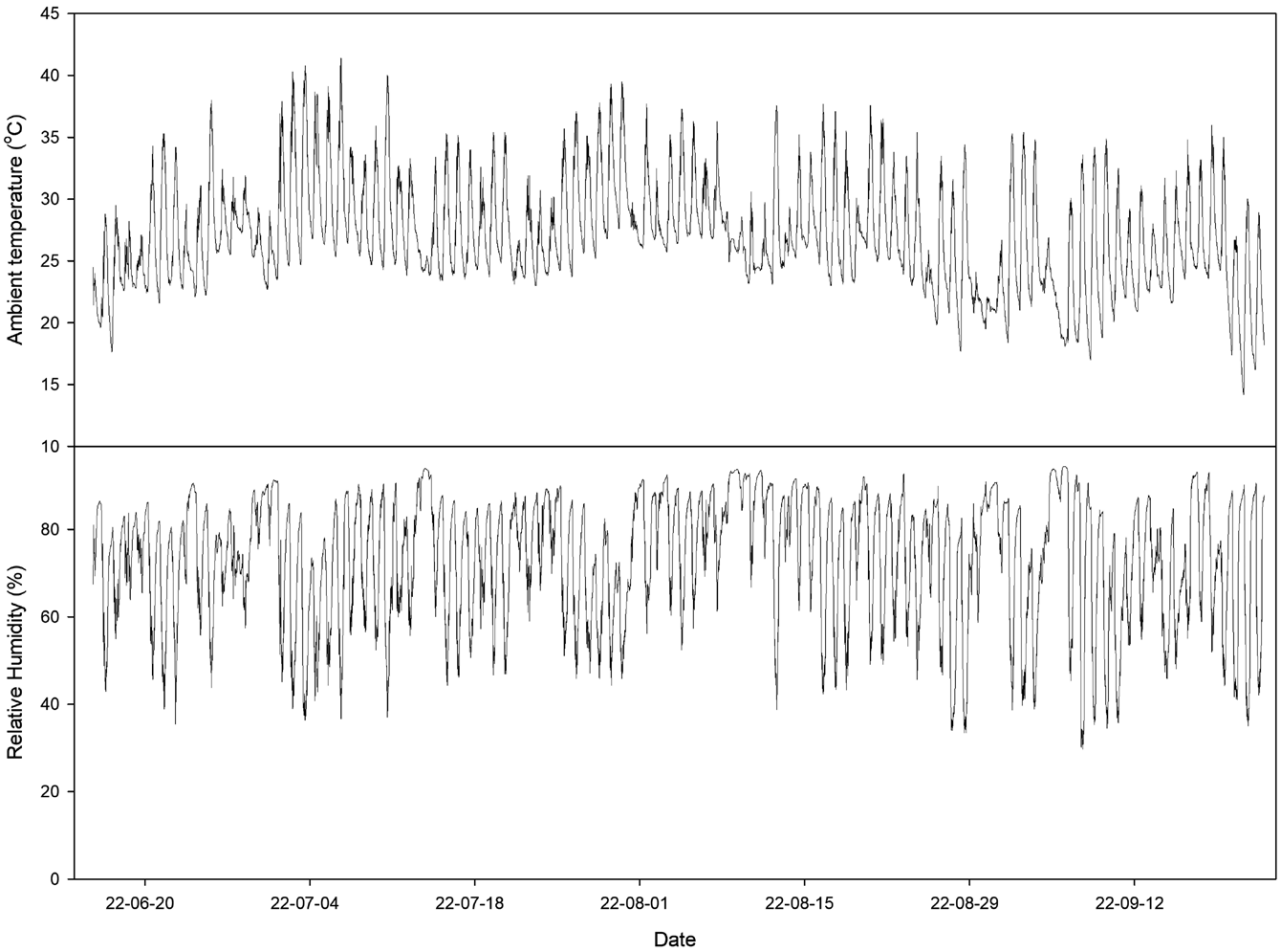
Ambient temperature and relative humidity inside greenhouse during cultivation during the summer season.

In the summer season, the paprika plants were grown with no cooling (NC), nutrient solution cooling (NSC) and the combination of NSC and substrate surround cooling (SSC) (NSC + SSC). In NSC, the solution temperature was set at 18 °C and maintained between 17.9 °C and 19.1 °C at daytime. In substrate surround cooling, the cooled water, whose temperature was set at 15 °C and maintained between 13.8 °C and 16.5 °C, was circulated when the ambient temperature inside the greenhouse was higher than 25 °C (Fig. 2) between 9:00 and 17:00. The cooling treatment was started at 11 July 2022, when the maximum day temperature exceeds the 40 °C for the first time and maintained during the whole cultivation periods (Fig. 3).

**Figure 4.**
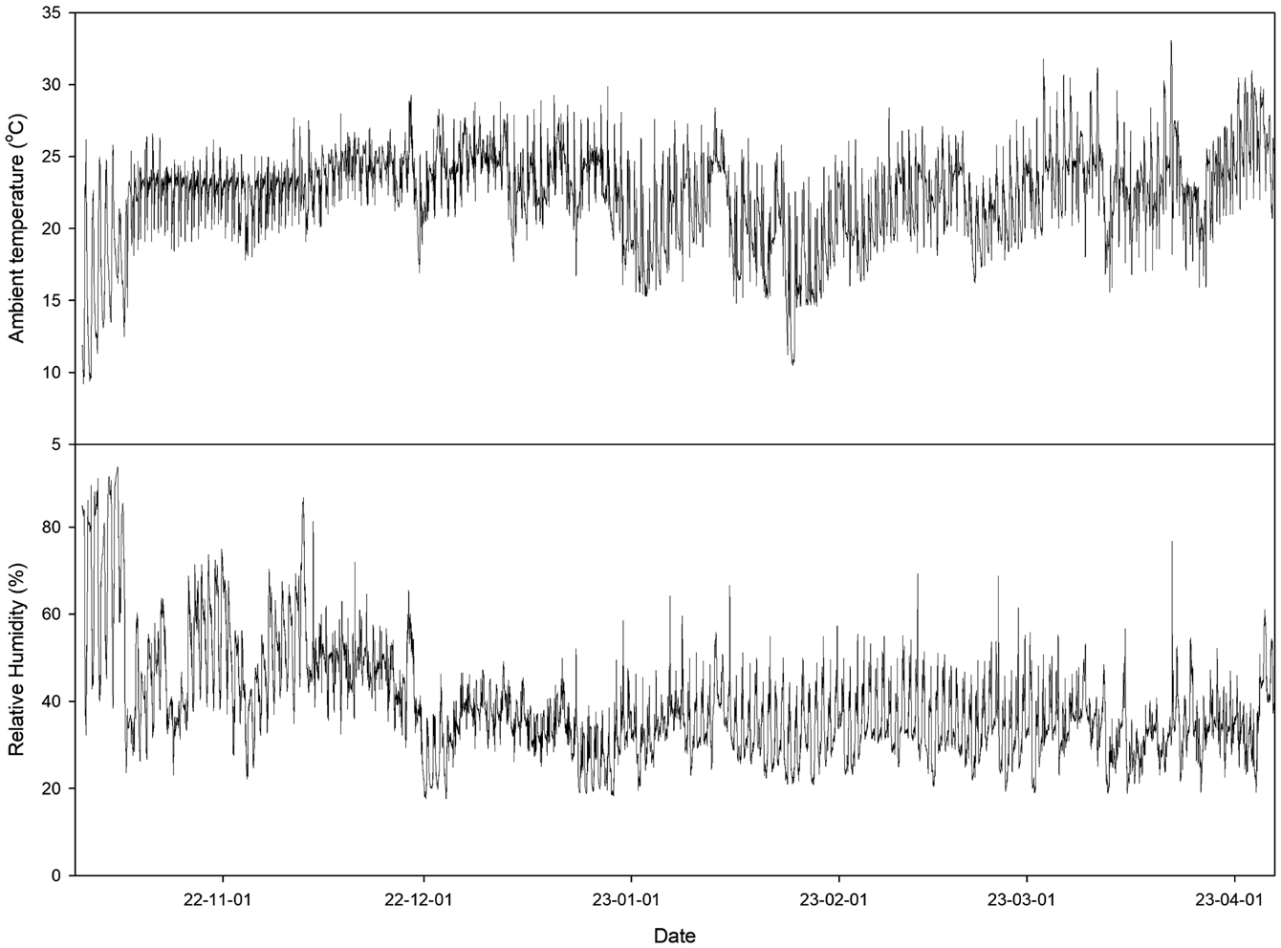
Ambient temperature and relative humidity inside greenhouse during cultivation during the winter season.

In the winter season, the paprika plants were grown with no heating treatment (NH), nutrient solution heating (NSH) and the combination of NSH and substrate surround heating (SSH) (NSH + SSH). In NSH, the tank temperature was set at 25 °C and maintained between 20.2 °C and 22.5°C, and in substrate surround heating, the heated water, which temperature was set at 30 °C and maintained between 25.5 °C and 33.6 °C, was circulated when the ambient temperature inside the greenhouse was lower than 25 °C at 6:00 to 8:00 and 16:00 to 20:00 (Fig. 2). The heating treatment was started at 27 December 2022, when the minimum night temperature falls below 15 °C for the first time, and maintained during the whole cultivation periods (Fig. 4).

### Evaluated parameters

The growth measurements for the cooling and heating experiments were performed on 23 September 2022 and 6 April 2023, respectively. Stem length and diameter, node number, leaf number, fresh and dry weights of leaf, stem and root, total leaf area and SPAD value were measured on 23 September 2022 [see Supporting Information—Table S1] and 6 April 2023 [see Supporting Information—Table S2]. The dry weights were measured after drying in an oven at 80 °C for a week. The total leaf area was measured with a leaf area metre (Li-3100, Li-Cor, Lincoln, NE, USA). The SPAD value was averaged with four leaves from the third newest unfolded leaf by chlorophyll metre (SPAD 502, Konica Minolta, Sakai, Japan). Fruits were harvested every week, and the weight of the fruit did not cause any physical disorder [see Supporting Information—Tables S3 and S4].

The drainage solutions were collected per treatment during the day while the temperature control system operated [see Supporting Information—Tables S5 and S6]. The collected drainage was filtered by a 0.20 μm microbial filter (Sartorius Minisart, Hannover, Germany), and then the nutrient composition, such as NH_4_-N and NO_3_-N, was investigated with ion chromatograph (ICS-3000, Dionex, CA, USA), and Mg^2+^, Total P (T-P), K^+^, Ca^2+^ and Total S (T-S) were investigated with ICP mass spectrometer (Varian 820-MS, Varian, CA, USA).

### Nutrient solution and substrate temperature sensing and energy usage calculation

T-type thermocouples measured the temperature of coir slabs, circulated water and the nutrient solution, and a data logger (CR1000X, Campbell Scientific Co., Logan, UT, USA) collected the data. The heat capacity of the coir slab and the temperature of the circulated water and substrate were used to calculate the system’s energy usage and efficiency.


$$\begin{array}{l} c_{slab}=\frac{c_{coir}(W_{coir})+c_{water}(W_{slab}-W_{coir})}{W_{slab}}, \end{array}$$

$$\begin{array}{l} E_{circulate}=(t_{inlet}-t_{outlet})c_{water}V_{circulate}, \end{array}$$

$$\begin{array}{l} E_{substrate}=(\Delta t_{SS}-\Delta t_{N})c_{coir}W_{slab}, \end{array}$$

$$\begin{array}{l} \eta_{SS}=\left(\frac{E_{substrate}}{E_{circulate}}\right)\times 100\%, \end{array}$$

where *c*_slab_ is the specific heat of coir slab saturated with water (cal g^−1^ °C^−1^); *c*_coir_ is the specific heat of dried coir; *c*_water_ is the specific heat of water; *W*_slab_ is the weight of coir slab saturated with water (g); *W*_coir_ is the weight of dried coir slab; *t*_inler_ is the circulate water temperature at the inlet of substrate surround system (°C); *t*_outlet_ is the circulate water temperature at the outlet of substrate surround system; *t*_ss_ is the temperature of substrate at nutrient solution and substrate surround temperature control treatment; *t*_N_ is the temperature of substrate with no temperature control; *E*_circulate_ is the energy use of circulate water at substrate surround temperature control system (J); *E*_substrate_ is the energy consumed by substrate with substrate surround temperature control system; *V*_circulate_ is the volumetric flow rate of circulate water at substrate surround temperature control system (L min^−1^) and *η*_SS_ is the energy efficiency of substrate surround temperature control system (Table 1).

**Table 1.** Properties of coir slab and water used in energy usage calculation.

Properties	Symbol	Value
Coir slab dry mass	*W*_coir_	1375 g
Coir slab wet mass	*W*_slab_	9129 g
Coir-specific heat	*c*_coir_	0.62 cal g^−1^ °C^−1^
Water-specific heat	*c*_water_	1 cal g^−1^ °C^−1^

### Statistical analysis

All data were analysed using Statistical Analysis System (SAS) 9.4 software (SAS Institute Inc., Cary, NC, USA). ANOVA was performed among the treatments, and the treatments were subsequently ranked using Duncan’s (1955) multiple range test at *P* < 0.05.

## Results

### Nutrient solution and substrate temperature in the summer season

**Figure 5.**
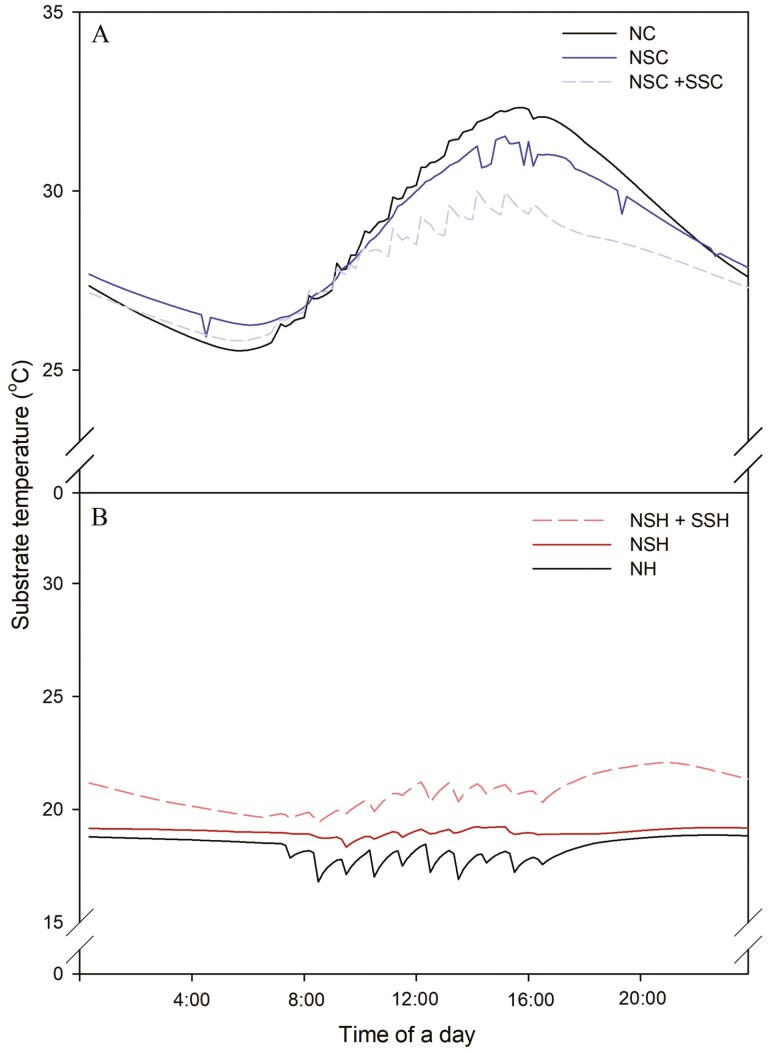
Average substrate temperature trend of Control and water circulation treatment in summer (A) and winter seasons (B).

In the summer season, the nutrient solution temperature of NC increased to 30 °C at noon (Fig. 5). With the NSC system, the temperature inside the nutrient solution tank was maintained between 18 °C and 24 °C. In NC, the substrate temperature increased with sunrise at 7:00 am, and the temperature rose to 32 °C at 4:00 pm, the highest of the day. In NSC and NSC + SSC treatment, the temperature pattern was similar to the NC, but the rate of increase decreased. The substrate temperature of NSC + SSC was maintained between 27 °C and 30 °C in the daytime, and the maximum temperature was 29.9 °C, about 2.4 °C lower than the NC. The substrate temperature of NSC also decreased compared to the control, even though the decrease was lower than that of NSC + SSC. The substrate temperature of NSC treatment is maintained between 26.9 °C and 29.9 °C in the daytime. At night-time, the substrate temperature of NSC + SSC was 2.8 °C higher than the Control at 17:00 when the treatment finished. The temperature difference between Control and NSC + SSC decreased rapidly during night-time, and the average substrate temperature between the two treatments was only 0.5 °C. Despite controlling the nutrient solution temperature, the substrate temperature rose temporarily every hour when the solution was irrigated by a heated solution that remained inside the pipe. It is important to note that the temperature measured at NSC can be lower than the average temperature of the substrate, and the cooling effect of NSC can be overestimated due to the location of the temperature sensor installed in the path of movement of the nutrient solution.

**Figure 6.**
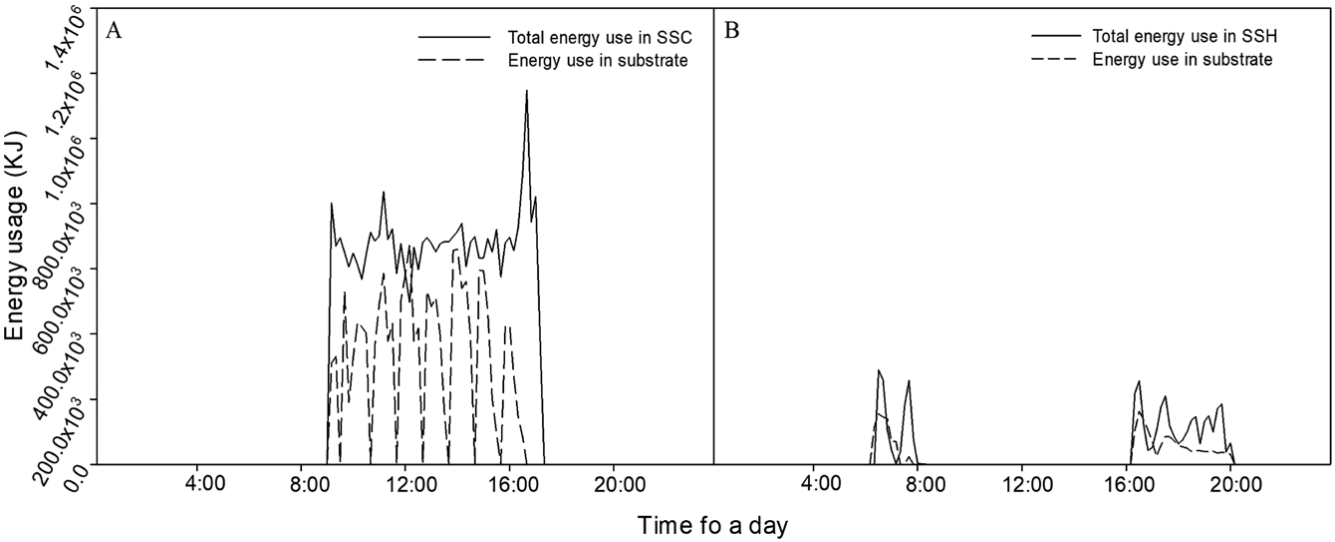
Average total energy usage in the water circulation system and energy consumption in the substrate in the summer (A) and winter seasons (B).

The temperature change of circulated water and substrate calculated energy usage of the water circulation system. The water circulated the substrate by an average flow rate of 26.38 kg min^−1^, and the average temperature of circulated water was 14.73 °C and 15.36 °C, at the inlet and outlet of the system, respectively, with the average difference being 0.6 °C. In the total amount of energy used in the NSC + SSC system, 48.4 % of energy was consumed by the coir slab and used to decrease the temperature (Fig. 6).

### Nutrient solution and substrate temperature in the winter season

In the winter season, the nutrient solution temperature of NH was maintained between 10 °C and 15 °C all day (Fig. 5). With the NSH system, the temperature inside the nutrient solution tank was maintained between 20 °C and 23 °C. The substrate temperature shows a similar tendency to nutrient solution temperature. The substrate temperature of NH was maintained between 16 °C and 19 °C. In NSH + SSH, the system increased the temperature, maintaining the substrate temperature between 19 °C and 23 °C all day. In the NSH + SSH treatment, the substrate temperature increased from 6:00 to 8:00 and 16:00 to 20:00 when the SSH system was running and the temperature was highest at 20:00 when direct before the treatment ended, which was 3.2 °C higher than control. The temperature control system also increases the night-time substrate temperature, even though the system does not activate at night. At NSH + SSH, the substrate temperature continuously decreases between sunset and sunrise, the substrate temperature was 1.1 °C higher than the control at 6:00, directly before the treatment starts.

When comparing the NSH and NH treatments, it is evident that the substrate temperature in NSH increased. This increase can be attributed to the steady temperature maintained by the NSH, which lowers the substrate temperature in NH. In the NH treatment, the temperature decreased at the irrigation timing due to the low temperature of the nutrient solution, whereas in the NSH treatment, the substrate temperature remained steady. However, it is important to note that the substrate temperature in the NSH treatment still fluctuated hourly when the nutrient solution was irrigated, similar to the NSC of the summer season. This suggests a potential overestimation of the heating effect of NSH, a point that warrants further investigation.

The water circulated the substrate at an average flow rate of 38.34 kg min^−1^, and the average temperature of the circulated water was 30.9 °C and 30.7 °C at the inlet and outlet of the system, respectively, with an average difference of 0.2 °C. Of the total energy used in the NSH + SSH system, 53.4 % was consumed by the coir slab to increase the temperature (Fig. 6).

### Growth in the summer season

In the summer season, the growth of paprika plants increased with cooling treatments, especially with the SSC system (Table 2). In contrast to the NSC treatment, which shows no significant difference in leaf growth, the leaf number increased significantly with decreased substrate temperature in the NSC + SSC treatment. Contrary to leaf number, leaf area and leaf fresh weights showed no significant difference between treatments. The parameters that are the indicators of stem growth, such as node number, stem length and stem dry weight, increased by substrate cooling treatment. The node number increased with both NSC and NSC + SSC, especially in NSC + SSC, and the difference was significant. The stem length and dry weight showed no significant difference in the NSC treatment. Contrary to the NSC treatment, in the NSC + SSC treatment, both parameters showed a significant difference. Similar to the above growth of paprika plants, the root growth also increased by cooling treatment. Root dry weight increased significantly with the NSC + SSC treatment. In the NSC treatment, the root dry weight shows no significant difference, although the weights increased compared to the NC.

**Table 2.** The stem length, stem diameter, nod number, leaf number, leaf area, stem, leaf root dry weight and SPAD value of paprika plants (*C. annuum* ‘Scirocco’) as affected by the cooling system 146 days after sowing in the greenhouse. Treatment means ± SD of six replicates. Means within each column and cultivar followed by the same letters are not significantly different according to Duncan’s multiple range test at *P* < 0.05. NC, no cooling; NSC, nutrient solution temperature control at 18 °C; NSC + SSC, beside NSC substrate surround cooling at a coolant temperature of 15 °C.

Treatment	Stem length (cm)	Stem diameter (mm)	Node number	Leaf number	Leaf area (cm^2^)	Dry weight			SPAD
						Stem (g)	Leaf (g)	Root (g)	
NC	161.3 ± 9.0 b	16.2 ± 1.3 ab	22.2 ± 1.6 b	55.3 ± 4.8 b	7619.8 ± 1044.4 a	58.2 ± 5.8 ab	42.7 ± 11.8 a	165.0 ± 42.5 b	55.7 ± 5.5 a
NSC	161.7 ± 5.6 b	15.4 ± 0.9 b	23.0 ± 1.4 ab	57.0 ± 2.8 ab	7818.8 ± 609.4 a	53.8 ± 8.8 b	44.1 ± 4.9 a	193.0 ± 61.5 ab	60.0 ± 2.8 a
NSC + SSC	170.5 ± 6.0 a	17.3 ± 1.0 a	24.2 ± 1.3 a	61.5 ± 3.6 a	8416.0 ± 1113.3 a	66.0 ± 6.2 a	50.2 ± 9.5 a	250.3 ± 71.9 a	52.7 ± 9.9 a

### Growth in the winter season

In the winter season, the growth of paprika plants increased with heating treatment, especially with the SSH system (Table 3). The leaf number and dry weight increased in NSH + SSH compared to the NH, which shows significant differences. The leaf area increased significantly by heating treatment in NSH and NSH + SSH, and the leaf area increased by 27.6 % and 50.7 %, respectively. Stem growth also increased by heating treatment. All parameters that indicate shoot growth, such as the node number, stem length and stem dry weight, increased at the NSH and NSH + SSH treatment compared to the NH. Contrary to the growth tendency of the above parts, root dry weight does not show any significant difference between treatments.

**Table 3.** The stem length, stem diameter, nod number, leaf number, leaf area, stem, leaf, root dry weight and SPAD value of paprika plants (*C. annuum* ‘Scirocco’) as affected by the heating system 204 days after sowing in the greenhouse. Treatment means ± SD of six replicates. Means within each column and cultivar followed by the same letters are not significantly different according to Duncan’s multiple range test at *P* < 0.05. NH, no heating; NSH, nutrient solution temperature control at 25 °C; NSH + SSH, beside NSH substrate surround heating at water temperature of 30 °C.

Treatment	Stem length (cm)	Stem diameter (mm)	Node number	Leaf number	Leaf area (cm^2^)	Dry weight			SPAD
						Stem (g)	Leaf (g)	Root (g)	
NH	120.0 ± 13.1 b	14.1 ± 0.5 a	24.5 ± 1.4 b	83.7 ± 4.3 b	5881.4 ± 866.3 c	25.4 ± 6.4 b	38.0 ± 7.7 b	182.2 ± 11.4 a	51.9 ± 5.9 a
NSH	151.2 ± 14.6 a	14.7 ± 1.2 a	27.8 ± 1.2 a	92.0 ± 8.0 a	7505.7 ± 1026.2 b	40.3 ± 10.6 a	42.1 ± 16.5 ab	196.6 ± 13.4 a	56.1 ± 3.3 a
NSH + SSH	155.3 ± 11.7 a	14.7 ± 1.4 a	28.0 ± 1.4 a	97.2 ± 3.4 a	8860.6 ± 915.7 a	42.2 ± 6.5 a	54.2 ± 5.9 ab	194.9 ± 12.3 a	55.2 ± 2.1 a

### Fruit yield in summer season

**Figure 7.**
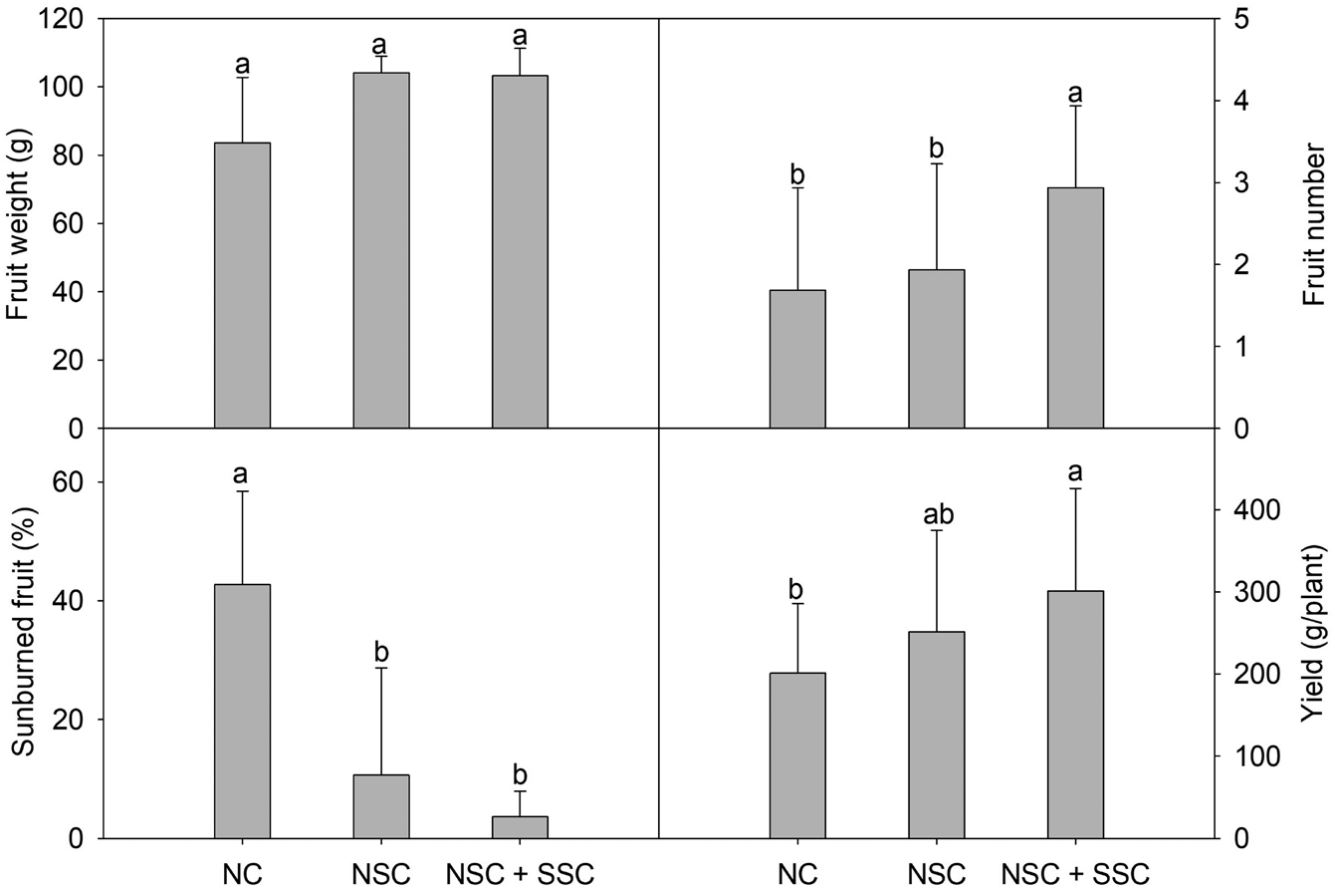
Mean fruit weight (A), fruit number (B), sunburned fruit rate (C) and total yield (D) of summer cultivated paprika with cooling treatment. The same letters above the bars indicate that means are not significantly different according to Duncan’s multiple range test at *P* < 0.05. Bars represent treatment means ± SD; *n* = 16 paprika plants.

In the summer, fruit yield increased by cooling treatment, especially the NSC + SSC treatment, showing significant differences by increasing 49.7 % of fruit yield compared to the NC. NSC also shows a 25 % increase in fruit yield compared to the NC, although there was no significant difference (Fig. 7). This difference was mainly caused by the mitigation of physiologically disordered fruit rate by the cooling system. In NC, a physiological disorder caused by poor Ca concentrations in fruits, such as sunburn and blossom-end rot, has occurred in 42.7 % of total fruit yield. The physiological disorder was suppressed by root-zone cooling systems to a rate of 10.6 % and 3.6 % at the NSC and NSC + SSC treatment, respectively. Fruit number also increased in NSC + SSC compared to the control.

### Fruit yield in the winter season

**Figure 8.**
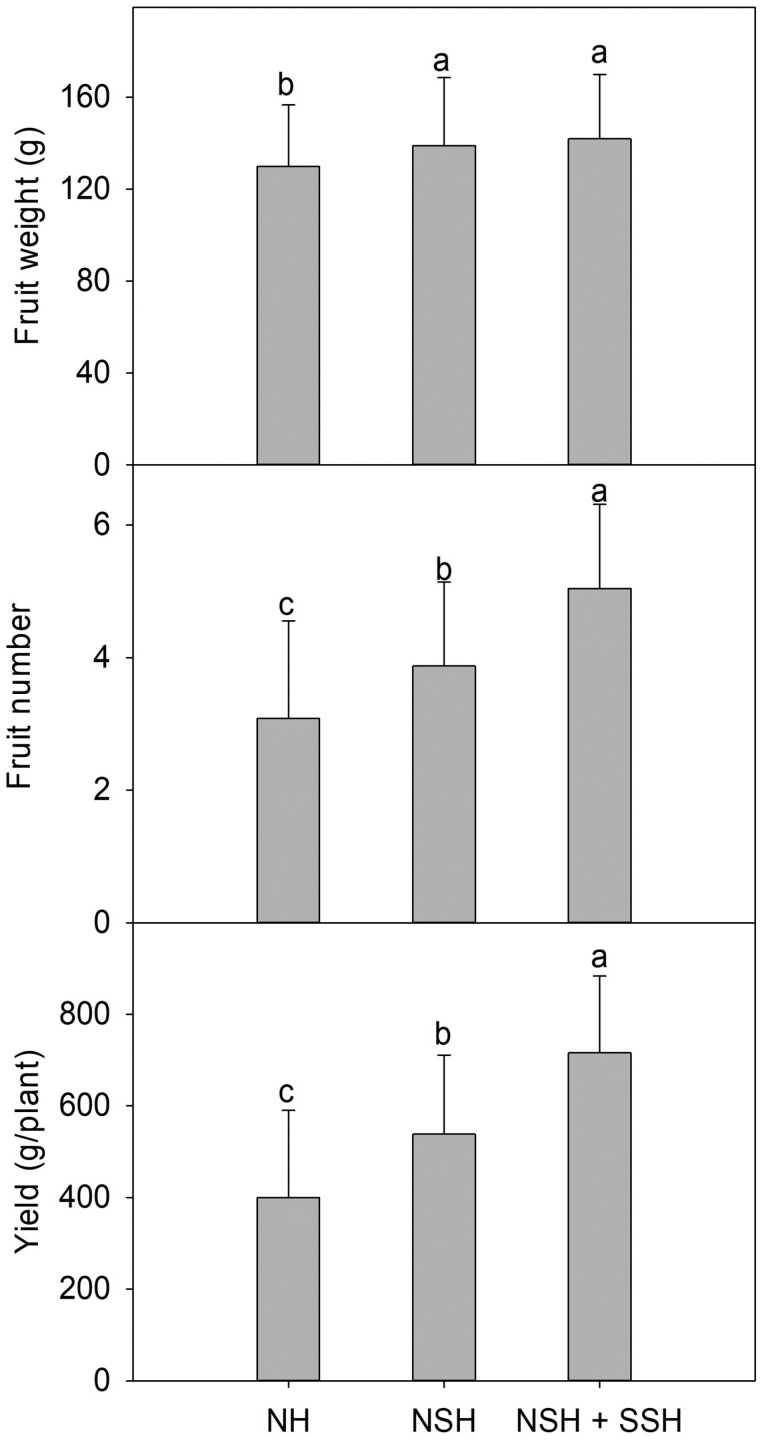
Mean fruit weight (A), fruit number (B) and total yield (C) of winter cultivated paprika with heating treatment. The same letters above the bars indicate that means are not significantly different according to Duncan’s multiple range test at *P* < 0.05. Bars represent treatment means ± SD; *n* = 24 paprika plants.

In the winter season, fruit numbers increased significantly by 25.6 % and 63.5 % in NSH and NSH + SSH, respectively (Fig. 8). Fruit weight also increased significantly by heating treatment. By increasing the fruit number and weight, yield increased significantly by 34.4 % and 78.7 % in NSH and NSH + SSH, respectively.

### Nutrient uptake in the summer season

**Figure 9.**
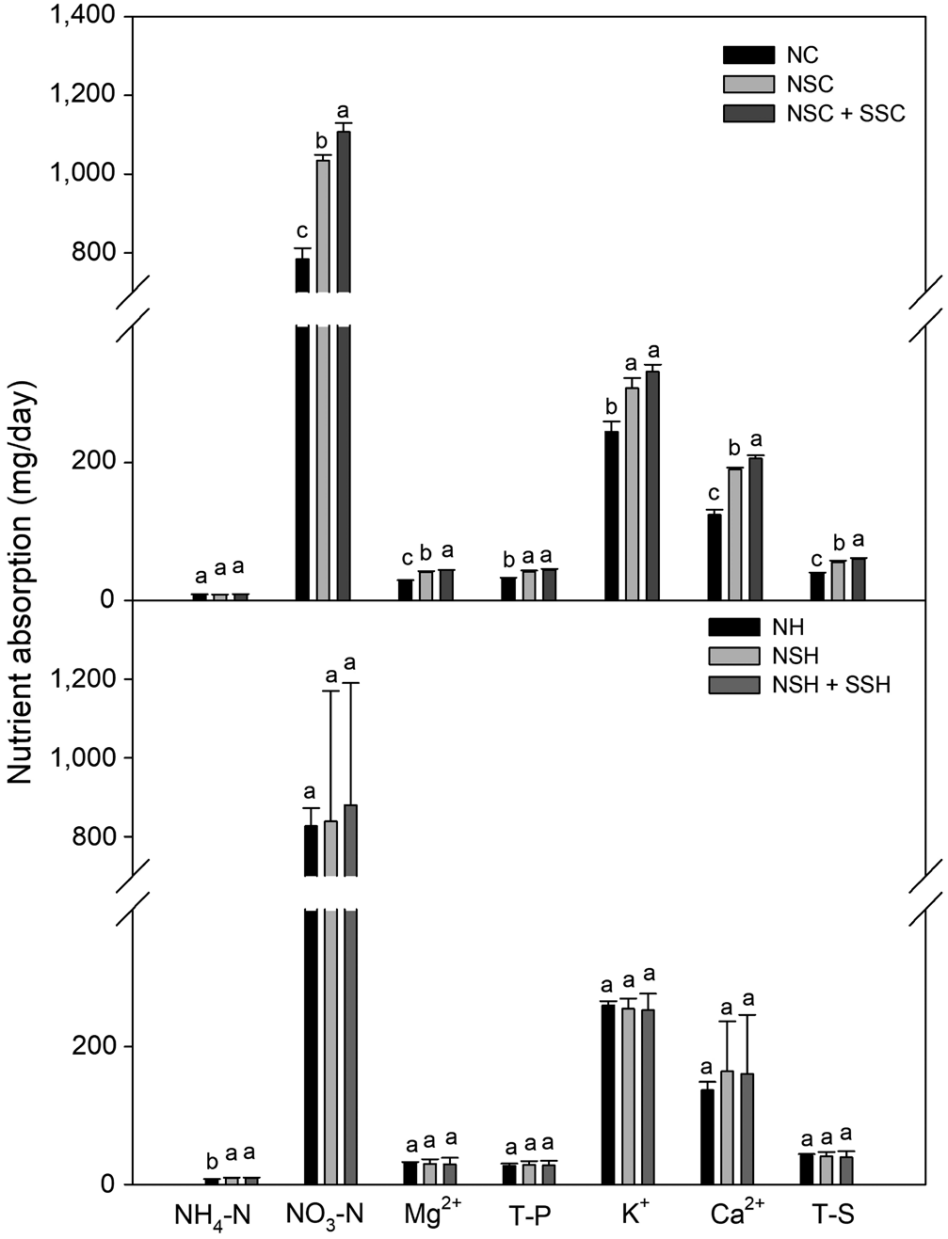
Mean daily nutrient absorption of NH_4_-N, NO_3_-N, Mg^2+^, Total P (T-P), K^+^, Ca^2+^ and Total S (T-S) in summer (A) and winter (B) cultivation with root-zone temperature control system. The same letters above the bars indicate that means are not significantly different according to Duncan’s multiple range test at *P* < 0.05. Bars represent treatment means ± SD; *n* = 3 days of drainage solution.

Nutrient solution uptake shows a difference by temperature control, mainly during the summer (Fig. 9). In the summer, all nutrient components except NH_4_-N showed a significant increase in absorption by cooling treatment. Absorption of T-P and K^+^ shows significant differences between the NC and cooling treatment, but there were no significant differences between the two cooling treatments, NSC and NSC + SSC. Absorption of NO_3_-N, Mg^2+^, Ca^2+^ and T-S shows significant differences amongst all the treatments. Especially for Ca^2+^, whose deficiency can cause physiological disorders in paprika fruit, such as sunburn and blossom-end rot (Alexander and Clough 1998; Paradiković *et al.* 2004; Kowalska and Sady 2012; Michałojć and Dzida 2012), they were increased by 52.7 % and 65.7 % at the NSC and NSC + SSC treatment, respectively.

### Nutrient uptake in the winter season

In the winter season, nutrient absorption results showed different patterns from the summer (Fig. 9). Only NH_4_-N shows significant increases in nutrient absorption patterns by heating treatment over the nutrient components.

## Discussion

### Substrate temperature and energy usage

When the root-zone temperature control system operated, the substrate consumed only 48.4 % and 53.4 % of the used energy and changed the temperature in the summer and winter seasons, respectively. The rest of the energy dissipated to the air which can decrease or increase the ambient temperature locally near the substrate. In the case of paprika, which has a specific phenotype that the temperature-sensitive organs such as shoot meristem, flower and fruit do not exist near the substrate, the effect of temperature change near the substrate seems negligible. To increase the energy portion consumed by the substrate, which can lead to an increase in energy usage, it is necessary to consider the insulation and the heat transfer rate of the square pipe.

In substrate surround temperature control, the water temperature changed by 0.63 °C and 0.2 °C in summer and winter, respectively. This small difference between the inlet and outlet temperatures can help to maintain the effect of the temperature control system equally in each substrate. When scaling up the system for installation in a commercial-size greenhouse, this temperature difference can be larger and make the temperature control effect unbalanced by the position of the substrate. To ensure the stability of the temperature control system, the circulation water flow rate must be increased by the size of the greenhouse where the system is planned to be installed.

### Growth and nutrient uptake in the summer season

In the summer season, root-zone cooling increased overall growth (Table 2). The high root-zone temperature, caused by high air temperature, can reduce the root viability of paprika (Choi *et al.* 2014). The electron leakage level, an indicator of root heat stress, was higher with high root-zone temperatures (Lee *et al.* 2022). The root viability of paprika can increase when the substrate temperature is controlled into the optimal range. Not just for the root-zone temperature, the heat stress above-ground that can cause the growth deficit can be reduced by root-zone temperature cooling. Lee *et al.* (2022) reported that the paprika seedlings’ growth and photosynthetic rate were increased by root-zone cooling, relieving the heat stress caused by the high ambient air temperature. The increased nutrient uptake by maintaining the root-zone temperature into the optimal range can also increase overall plant growth (Fig. 9). In tomatoes, the nutrient uptake critically decreases when the substrate temperature increases over 26.7 °C (Tindall *et al.* 1990). Even though the substrate temperature is maintained in the optimal range, the high nutrient solution temperature can decrease the nutrient uptake by reducing the solubility of fertilizer and the uptake capacity of the root (Trejo-Téllez and Gómez-Merino 2012).

### Growth and nutrient uptake in the winter season

In the winter season, the above-ground part growth of paprika increased by root-zone heating (Table 3). The effect of root-zone heating on growth promotion was reported on tomato and paprika (Abdel-Mawgoud *et al.* 2005; Kawasaki *et al.* 2014). Similar to the effect of increased air temperature in the early morning that promotes paprika growth against the freezing effects (Ghosal and Tiwari 2004), root-zone heating also increases the leaf and stem dry weights. The elevated root-zone temperature in the early morning increases the water uptake by increasing root activity and transpiration and can promote plant growth (Abdel-Mawgoud *et al.* 2005). The substrate temperature differences in the winter season were not enough to show a significant difference in nutrient solution uptake except NH_4_-N (Fig. 9). Also, the result of the study about the substrate temperature and nutrient uptake in tomatoes shows that the macronutrient uptake with substrate temperature between 18.3 °C and 22.8 °C, which is the daily average temperature of NH and NSH + SSH, does not show significant difference except that of NH_4_-N (Tindall *et al.* 1990).

### Fruit yield in the summer season

In the summer season, physiological disorders such as sunburn and blossom-end rot occurred at Control and NSC treatment (Fig. 7). Sunburn and blossom-end rot are main fruit physiological disorders in Solanaceae family like tomatoes and peppers, whose occurrence rates increase linearly with increasing temperature (Adams and Ho 1993; Maughan *et al.* 2017). As the substrate temperature increase exceeds the optimum range, the nutrient uptake capacity of the root decreases (Tindall *et al.* 1990) by decreasing water uptake (Feng *et al.* 1990) and root respiration (Gliński and Lipiec 1990), the paprika plant shows the same decreased tendency for nutrient absorption, including Ca, with increasing substrate temperature (Fig. 9).

The poor absorption of Ca can decrease the strength of the fruit surface and increase the occurrence rate of sunburn and blossom-end rot (Paradiković *et al.* 2004). As discussed above, the number of physiologically disordered fruits increased with a decrease in Ca absorption. The fruit number also increased with decreasing substrate temperature. High-temperature stress, which can have deleterious effects on flower development and cause flower abscission (Sugiyama *et al.* 1965; Jie and Kong 1998), can be alleviated by maintaining the root-zone temperature within an optimal range (Kuroyanagi and Paulsen 1988; Sasaki and Itagi 1989). In addition, Kwack *et al.* (2014) reported that flower development was promoted by substrate cooling of paprika seedlings.

### Fruit yield in the winter season

In the winter season, the fruit number and weight increased with heating treatment (Fig. 8). The increase in fruit number by heating was reported frequently on tomatoes (Maher 1978; Kageyama 1980; Gosselin and Trudel 1983), contrary to these the flowering number dose does not show any increasing tendency by nutrient solution or substrate heating (Gosselin and Trudel 1983). Paprika shows the same tendency as tomatoes, with an increase in fruit number but no differences in flowering number, indicating that the increase may be due to a higher percentage of fruit set (Abdel-Mawgoud *et al.* 2005). The increasing fruit weight may be due to a better plant water status and higher assimilate production caused by increasing transpiration and leaf area, respectively (Ali *et al.* 2000; Abdel-Mawgoud *et al.* 2005). Gosselin and Trudel (1983) also reported that paprika plants with low substrate temperature produced small, misshapen fruits by rapidly developing red pigmentation.

## Conclusions

This study highlights the effects of root-zone temperature control systems on promoting growth and increasing the yield of paprika. Both in summer and winter, the root-zone temperature systems help to maintain the root-zone temperature in the optimal range, even though the ambient air temperature has fluctuated. In the summer, root and shoot dry weights increased with root-zone cooling due to increased water and nutrient uptake. The shoot dry weight increases in the winter, even though the root dry weights do not differ significantly. In both seasons, the fruit yield increased by root-zone temperature control. The fruit number increased gradually with the addition of a root-zone temperature control system in both seasons. The heat pump-type root-zone heating can be considered a novel temperature control system that enhances yield with high energy efficiency.

## Supporting Information

The following additional information is available in the online version of this article –

Table S1. Raw data of plant growth as affected by the cooling system 146 days after sowing in the greenhouse.

Table S2. Raw data of plant growth as affected by the heating system 204 days after sowing in the greenhouse.

Table S3. Raw data of fruit yield in summer cultivation.

Table S4. Raw data of fruit yield in winter cultivation.

Table S5. Raw data of nutrient absorption in summer cultivation.

Table S6. Raw data of nutrient absorption in winter cultivation.

plae047_suppl_Supplementary_Materials

## Sources of Funding

This work was supported by Korea Institute of Planning and Evaluating for Technology in Food, Agriculture and Forestry (IPET) and Korea Smart Farm R&D Foundation (KosFarm) through Smart Farm Innovation Technology Development Program, funded by Ministry of Agriculture, Food and Rural Affairs (MAFRA) and Ministry of Science and ICT (MSIT), Rural Development Administration, Republic of Korea [grant number 421041-03].

## Data Availability

The data underlying this article are available in supporting information. The other data analysed are included in this manuscript.
