# Supplementary material for: Development of a root-zone temperature control system using air-source heat pump and its impact on the growth and yield of paprika
Source: AoB Plants. 2024 Sep 14;16(5):plae047. doi: 10.1093/aobpla/plae047 (PMC11489771; doi:10.1093/aobpla/plae047)
Supplement: plae047_suppl_Supplementary_Materials [file plae047_suppl_supplementary_materials.docx]

| Treatment | Stem length  (cm) | Stem diameter  (mm) | Nod number | Leaf number | Stem dry weight  (g) | Leaf dry weight  (g) | Root dry weight  (g) | Leaf area  (cm^2^) | SPAD |
| --- | --- | --- | --- | --- | --- | --- | --- | --- | --- |
| Control | 172 | 18 | 24 | 64 | 61.75 | 65.88 | 155.37 | 9200.84 | 49.3 |
| Control | 153 | 15.24 | 24 | 55 | 40.64 | 60 | 125.67 | 7674.28 | 49.3 |
| Control | 165 | 15.39 | 22 | 53 | 46.47 | 62.15 | 153.45 | 7370.1 | 58 |
| Control | 164 | 15.51 | 20 | 53 | 45.34 | 57.98 | 121.89 | 7769.41 | 56.1 |
| Control | 166 | 17.56 | 22 | 50 | 35.54 | 50.84 | 214.71 | 5933.34 | 63.4 |
| Control | 148 | 15.28 | 21 | 57 | 26.54 | 52.43 | 219.14 | 7770.68 | 58 |
| NSC | 153 | 14.09 | 25 | 61 | 45.57 | 55.83 | 244.77 | 8697.68 | 61.1 |
| NSC | 161 | 15.7 | 21 | 56 | 51.17 | 54.93 | 144.18 | 7598.64 | 57.6 |
| NSC | 167 | 16.63 | 22 | 53 | 39.46 | 57.23 | 146.92 | 7186.56 | 63.1 |
| NSC | 168 | 15.05 | 23 | 57 | 44.59 | 50.45 | 121.46 | 7687.63 | 59.6 |
| NSC | 158 | 15.27 | 23 | 59 | 37.7 | 39 | 251.58 | 7631.32 | 56 |
| NSC | 163 | 15.89 | 24 | 56 | 46.29 | 65.6 | 249.23 | 6911.02 | 62.7 |
| NSC+SSC | 162 | 16.79 | 22 | 61 | 42.97 | 59.66 | 259.57 | 7478.93 | 52.9 |
| NSC+SSC | 179 | 17.42 | 24 | 56 | 44.87 | 67.11 | 288.13 | 7604.87 | 53.9 |
| NSC+SSC | 174 | 18.25 | 25 | 61 | 63.47 | 75.63 | 221.39 | 9455.53 | 58.9 |
| NSC+SSC | 172 | 15.62 | 24 | 65 | 54.88 | 68.4 | 365.31 | 10009.84 | 56.8 |
| NSC+SSC | 166 | 18.01 | 26 | 60 | 38.68 | 58.62 | 157.24 | 7404.2 | 60.3 |
| NSC+SSC | 170 | 17.5 | 24 | 66 | 56.24 | 66.34 | 209.96 | 8542.48 | 33.3 |

Table S1. Raw data of stem length, stem diameter, nod number, leaf number, leaf area, stem, leaf, and root dry weight, and SPAD value of paprika plants (*Capsicum annuum L. ‘Scirocco’*) as affected by the cooling system 146 days after sowing in the greenhouse.

| Treatment | Stem length  (cm) | Stem diameter  (mm) | Nod number | Leaf number | Stem dry weight  (g) | Leaf dry weight  (g) | Root dry weight  (g) | Leaf area  (cm^2^) | SPAD |
| --- | --- | --- | --- | --- | --- | --- | --- | --- | --- |
| Control | 131 | 15.07 | 26 | 83 | 29.83 | 45.63 | 182.7 | 6707.59 | 59.1 |
| Control | 135 | 14.01 | 26 | 83 | 30.82 | 40.5 | 196.0 | 6030.35 | 53.3 |
| Control | 129.5 | 14.3 | 25 | 85 | 32.68 | 33.73 | 185.0 | 5502.25 | 52.6 |
| Control | 110.6 | 13.72 | 24 | 88 | 19.08 | 47.49 | 178.7 | 6904.76 | 50.4 |
| Control | 108 | 13.62 | 23 | 87 | 20.35 | 31.99 | 188.7 | 5593.75 | 54.7 |
| Control | 106 | 13.82 | 23 | 76 | 19.44 | 28.63 | 162.3 | 4549.7 | 41.4 |
| NSH | 139 | 14.73 | 28 | 90 | 40.35 | 48.39 | 179.7 | 7082.82 | 53.4 |
| NSH | 158.5 | 15.54 | 28 | 99 | 39.19 | 54.49 | 207.3 | 8833.92 | 58 |
| NSH | 156.5 | 15 | 27 | 90 | 38.02 | 44.02 | 179.3 | 7286.08 | 50.6 |
| NSH | 174.5 | 15.98 | 30 | 104 | 59.12 | 58.55 | 207.0 | 8715.66 | 57.8 |
| NSH | 141.5 | 14.2 | 29 | 86 | 26.31 | 13.31 | 204.0 | 6639.43 | 59.3 |
| NSH | 137 | 12.46 | 25 | 83 | 38.91 | 33.77 | 202.0 | 6476.43 | 57.2 |
| NSH+SSH | 164 | 15.95 | 32 | 93 | 41.24 | 53.48 | 204.3 | 8480.5 | 53.2 |
| NSH+SSH | 169 | 12.49 | 29 | 100 | 48.3 | 60.07 | 178.7 | 10010.2 | 54 |
| NSH+SSH | 145.5 | 15.63 | 26 | 102 | 47.12 | 60.62 | 192.7 | 9653.59 | 54.1 |
| NSH+SSH | 162.5 | 16.13 | 29 | 97 | 47.39 | 53.65 | 187.7 | 8561.34 | 56.1 |
| NSH+SSH | 139 | 13.69 | 24 | 97 | 32.99 | 44.54 | 192.7 | 7451.02 | 59 |
| NSH+SSH | 152 | 14.47 | 28 | 94 | 36.01 | 52.89 | 213.3 | 9006.72 | 54.9 |

Table S2. Raw data of stem length, stem diameter, nod number, leaf number, leaf area, stem, leaf, and root dry weight, and SPAD value of paprika plants (*Capsicum annuum L. ‘Scirocco’*) as affected by the heating system 204 days after sowing in the greenhouse.

Table S3. Raw data of mean fruit weight, fruit number, sunburned fruit rate, and total yield in summer cultivation.

| Treatment | Fruit number | Average fruit  weight (g) | Sunburned fruit  (%) | Yield  (g) |
| --- | --- | --- | --- | --- |
| Control | 2 | 95.2 | 33 | 190.4 |
| Control | 0 | 0 | 0 | 0.0 |
| Control | 4 | 113.2 | 0 | 452.8 |
| Control | 2 | 97.5 | 0 | 194.9 |
| Control | 1 | 85.7 | 50 | 85.7 |
| Control | 3 | 96.0 | 0 | 287.9 |
| Control | 0 | 118.6 | 0 | 0.0 |
| Control | 1 | 91.9 | 50 | 91.9 |
| Control | 2 | 103.7 | 0 | 207.4 |
| Control | 4 | 98.8 | 50 | 395.2 |
| Control | 2 | 0.0 | 100 | 0.0 |
| Control | 0 | 100.2 | 50 | 0.0 |
| Control | 1 | 99.0 | 0 | 99.0 |
| Control | 2 | 0.0 | 100 | 0.0 |
| Control | 1 | 110.0 | 50 | 110.0 |
| Control | 2 | 127.6 | 0 | 255.2 |
| NSC | 1 | 118.3 | 50 | 118.3 |
| NSC | 2 | 102.7 | 0 | 205.4 |
| NSC | 2 | 118.3 | 0 | 236.5 |
| NSC | 1 | 109.1 | 0 | 109.1 |
| NSC | 3 | 110.1 | 0 | 330.2 |
| NSC | 3 | 86.1 | 0 | 258.3 |
| NSC | 1 | 118.6 | 0 | 118.6 |
| NSC | 4 | 93.7 | 0 | 374.6 |
| NSC | 4 | 118.5 | 33 | 473.8 |
| NSC | 3 | 92.1 | 67 | 276.3 |
| NSC | 1 | 69.3 | 0 | 69.3 |
| NSC | 0 | 103.8 | 20 | 0.0 |
| NSC | 1 | 90.4 | 0 | 90.4 |
| NSC | 2 | 112.2 | 0 | 224.4 |
| NSC | 0 | 109.7 | 0 | 0.0 |
| NSC | 3 | 113.0 | 0 | 339.1 |
| NSC+SSC | 3 | 118.7 | 0 | 356.0 |
| NSC+SSC | 3 | 111.6 | 0 | 334.9 |
| NSC+SSC | 3 | 107.1 | 0 | 321.2 |
| NSC+SSC | 4 | 84.9 | 0 | 339.6 |
| NSC+SSC | 3 | 127.4 | 0 | 382.1 |
| NSC+SSC | 1 | 88.2 | 0 | 88.2 |
| NSC+SSC | 2 | 117.9 | 0 | 235.8 |
| NSC+SSC | 3 | 131.8 | 0 | 395.3 |
| NSC+SSC | 4 | 115.7 | 0 | 462.9 |
| NSC+SSC | 4 | 112.0 | 33 | 448.0 |
| NSC+SSC | 3 | 106.9 | 0 | 320.7 |
| NSC+SSC | 3 | 87.8 | 0 | 263.4 |
| NSC+SSC | 5 | 98.1 | 25 | 490.5 |
| NSC+SSC | 2 | 82.1 | 0 | 164.2 |
| NSC+SSC | 2 | 94.6 | 0 | 189.2 |
| NSC+SSC | 2 | 67.7 | 0 | 135.4 |

Table S4. Raw data of mean fruit weight, fruit number, and total yield in winter cultivation

| Treatment | Fruit number | Average fruit  weight (g) | Yield  (g) |
| --- | --- | --- | --- |
| Control | 5 | 115.5 | 577.7 |
| Control | 0 | 0 | 0 |
| Control | 5 | 110.3 | 551.4 |
| Control | 3 | 124.0 | 371.9 |
| Control | 1 | 169.7 | 169.7 |
| Control | 3 | 108.2 | 324.6 |
| Control | 5 | 121.6 | 607.8 |
| Control | 4 | 137.0 | 548.0 |
| Control | 2 | 157.0 | 314.0 |
| Control | 4 | 128.9 | 515.8 |
| Control | 3 | 131.5 | 394.6 |
| Control | 2 | 136.4 | 272.9 |
| Control | 2 | 89.9 | 179.8 |
| Control | 4 | 137.2 | 548.9 |
| Control | 4 | 134.9 | 539.8 |
| Control | 2 | 113.3 | 226.7 |
| Control | 2 | 134.7 | 269.3 |
| Control | 5 | 131.6 | 657.8 |
| Control | 4 | 132.9 | 531.5 |
| Control | 4 | 162.1 | 648.4 |
| Control | 0 | 0 | 0 |
| Control | 4 | 115.6 | 462.5 |
| Control | 3 | 146.3 | 439.0 |
| Control | 3 | 152.0 | 456.0 |
| Control | 5 | 121.8 | 608.8 |
| NSH | 2 | 163.7 | 327.4 |
| NSH | 5 | 116.7 | 583.4 |
| NSH | 5 | 111.1 | 555.7 |
| NSH | 5 | 151.7 | 758.7 |
| NSH | 4 | 146.8 | 587.2 |
| NSH | 5 | 156.6 | 782.8 |
| NSH | 4 | 149.0 | 595.9 |
| NSH | 3 | 127.6 | 382.7 |
| NSH | 4 | 112.5 | 449.9 |
| NSH | 5 | 139.0 | 694.9 |
| NSH | 4 | 129.9 | 519.5 |
| NSH | 4 | 139.2 | 556.8 |
| NSH | 4 | 121.5 | 485.9 |
| NSH | 1 | 158.4 | 158.4 |
| NSH | 3 | 162.2 | 486.7 |
| NSH | 7 | 137.0 | 958.8 |
| NSH | 3 | 94.8 | 284.5 |
| NSH | 3 | 141.7 | 425.0 |
| NSH | 4 | 161.9 | 647.6 |
| NSH | 2 | 181.5 | 363.0 |
| NSH | 4 | 140.6 | 562.3 |
| NSH | 3 | 162.5 | 487.5 |
| NSH | 4 | 163.4 | 653.6 |
| NSH | 6 | 131.7 | 790.3 |
| NSH+SSH | 5 | 158.2 | 791.0 |
| NSH+SSH | 5 | 129.8 | 648.9 |
| NSH+SSH | 5 | 132.7 | 663.3 |
| NSH+SSH | 6 | 136.4 | 818.1 |
| NSH+SSH | 3 | 162.3 | 486.8 |
| NSH+SSH | 5 | 134.3 | 671.7 |
| NSH+SSH | 5 | 152.428 | 762.14 |
| NSH+SSH | 5 | 132.38 | 661.9 |
| NSH+SSH | 6 | 139.6617 | 837.97 |
| NSH+SSH | 4 | 148.615 | 594.46 |
| NSH+SSH | 6 | 148.215 | 889.29 |
| NSH+SSH | 7 | 139.3786 | 975.65 |
| NSH+SSH | 6 | 135.6817 | 814.09 |
| NSH+SSH | 5 | 133.622 | 668.11 |
| NSH+SSH | 2 | 176.62 | 353.24 |
| NSH+SSH | 8 | 147.1688 | 1177.35 |
| NSH+SSH | 4 | 136.8425 | 547.37 |
| NSH+SSH | 5 | 135.48 | 677.4 |
| NSH+SSH | 4 | 154.6925 | 618.77 |
| NSH+SSH | 4 | 145.57 | 582.28 |
| NSH+SSH | 4 | 153.3 | 613.2 |
| NSH+SSH | 5 | 143.954 | 719.77 |
| NSH+SSH | 6 | 134.775 | 808.65 |

| Treatment | NH_4_-Ｎ | NO_3_-Ｎ | Mg^2+^ | T-P | K^+^ | Ca^2+^ | T-S |
| --- | --- | --- | --- | --- | --- | --- | --- |
| Control | 8.5 | 807.4 | 29.6 | 31.2 | 250.9 | 125.5 | 40.6 |
| Control | 8.9 | 752.7 | 28.2 | 31.2 | 227.9 | 116.5 | 39.4 |
| Control | 7.8 | 791.9 | 28.5 | 33.2 | 256.1 | 131.4 | 39.3 |
| NSC | 8.2 | 1050.9 | 40.1 | 40.2 | 294.8 | 191.3 | 53.3 |
| NSC | 8.7 | 1025.6 | 41.3 | 41.4 | 307.4 | 186.7 | 55.5 |
| NSC | 7.9 | 1026.1 | 42.4 | 43.5 | 323.5 | 192.4 | 57.7 |
| NSC+SSC | 8.9 | 1133.0 | 44.3 | 43.0 | 323.4 | 208.0 | 59.2 |
| NSC+SSC | 9.6 | 1097.0 | 44.1 | 44.1 | 330.4 | 201.1 | 60.4 |
| NSC+SSC | 8.8 | 1092.1 | 44.4 | 46.0 | 343.5 | 209.8 | 61.6 |

Table S5. Raw data of nutrient absorption of NH_4_-N, NO_3_-N, Mg^2+^, Total P (T-P), K^+^, Ca^2+^, and Total S (T-S) in summer cultivation.

Table S6. Raw data of nutrient absorption of NH_4_-N, NO_3_-N, Mg^2+^, Total P (T-P), K^+^, Ca^2+^, and Total S (T-S) in winter cultivation.

| Treatment | NH_4_-Ｎ | NO_3_-Ｎ | Mg^2+^ | T-P | K^+^ | Ca^2+^ | T-S |
| --- | --- | --- | --- | --- | --- | --- | --- |
| Control | 8.3 | 1067.3 | 40.0 | 35.8 | 318.1 | 181.6 | 54.0 |
| Control | 8.2 | 1025.9 | 42.1 | 39.3 | 309.6 | 195.6 | 56.7 |
| Control | 8.3 | 1003.1 | 40.1 | 35.7 | 312.6 | 180.1 | 53.6 |
| NSH | 9.3 | 1052.9 | 32.4 | 32.6 | 261.9 | 206.2 | 44.2 |
| NSH | 8.8 | 457.8 | 34.6 | 30.2 | 265.2 | 205.8 | 44.2 |
| NSH | 9.6 | 1006.2 | 22.1 | 22.0 | 238.2 | 80.6 | 33.7 |
| NSH+SSH | 9.6 | 1130.9 | 30.3 | 29.4 | 243.8 | 196.3 | 40.4 |
| NSH+SSH | 8.6 | 533.1 | 38.0 | 33.2 | 279.9 | 222.6 | 47.4 |
| NSH+SSH | 9.6 | 976.6 | 18.8 | 20.6 | 235.4 | 62.8 | 30.1 |
